# Artificial Intelligence in Food Bank and Pantry Services: A Systematic Review

**DOI:** 10.3390/nu17091461

**Published:** 2025-04-26

**Authors:** Yuanyuan Yang, Ruopeng An, Cao Fang, Dan Ferris

**Affiliations:** 1 Brown School, Washington University in St. Louis, St. Louis, MO 63130, USA; cao.f@wustl.edu (C.F.); dan.ferris@wustl.edu (D.F.); 2 Sliver School of Social Work, New York University, New York, NY 10003, USA

**Keywords:** food banks, food pantries, food services, artificial intelligence, data science, artificial intelligence ethics, systematic review

## Abstract

Background/Objectives: Food banks and pantries play a critical role in improving food security through allocating essential resources to households that lack consistent access to sufficient and nutritious food. However, these organizations encounter significant operational challenges, including variability in food donations, volunteer shortages, and difficulties in matching supply with demand. Artificial intelligence (AI) has become increasingly prevalent in various sectors of the food industry and related services, highlighting its potential applicability in addressing these operational complexities. Methods: This study systematically reviewed empirical evidence on AI applications in food banks and pantry services published before 15 April 2025. The review followed the Preferred Reporting Items for Systematic Reviews and Meta-Analyses (PRISMA) guidelines. A comprehensive keyword and reference search was conducted in 11 electronic bibliographic databases: PubMed, Web of Science, Scopus, MEDLINE, APA PsycArticles, APA PsycInfo, CINAHL Plus, EconLit with Full Text, Applied Science & Technology Full Text (H.W. Wilson), Family & Society Studies Worldwide, and SocINDEX. Results: We identified five peer-reviewed papers published from 2015 to 2024, four of which utilized structured data machine learning algorithms, including neural networks, K-means clustering, random forests, and Bayesian additive regression trees. The remaining study employed text-based topic modeling to analyze food bank and pantry services. Of the five papers, three focused on the food donation process, and two examined food collection and distribution. Discussion: Collectively, these studies show the emerging potential for AI applications to enhance food bank and pantry operations. However, notable limitations were identified, including the scarcity of studies on this topic, restricted geographic scopes, and methodological challenges such as the insufficient discussion of data representativeness and statistical power. None of the studies addressed AI ethics, including model bias and fairness, or discussed intervention and policy implications in depth. Further studies should investigate innovative AI-driven solutions within food banks and pantries to help alleviate food insecurity.

## 1. Introduction

Artificial intelligence (AI) has emerged as a transformative technology across various domains. In the food sector, AI technologies such as smart sensors, machine learning (ML), deep learning (DL), and fuzzy logic systems have been extensively applied to food quality assessment, inventory management, supply chain optimization, customer service, and nutrition [1,2,3].

According to Kumar et al. [4], the use of AI in the food sector can be categorized into two main classes: food quality management and food security management. For food quality and safety management, AI can employ advanced technologies for identification, classification, assessment, monitoring, and control. For example, image analysis integrated with machine learning has been used to develop real-time food freshness detection systems, reducing the consumption of substandard food products [5]. In food security management, Dolgui et al. [6] developed a mathematical model incorporating a novel genetic algorithm to optimize production, inventory, and distribution processes for perishable products. In a quasi-experimental study involving approximately 900 kitchens, Nu et al. [7] demonstrated that AI-driven image classification technology reduced food waste by 29%, with an additional 30% average reduction achieved by adopting computer vision-based automatic recognition systems. These applications highlight the transformative potential of AI in optimizing traditional food systems, enabling the food sector to enhance yields, quality, safety, and nutrition.

Globally, an estimated 28.9% of the population experienced moderate or severe food insecurity [8]. In developed countries, the reported rates of food insecurity are 10% in the UK, 15.9% in Canada, and 13.5% in the United States [9,10,11]. A food pantry is a community-based organization that directly provides free food to individuals and households experiencing food insecurity, relying heavily on donated food and volunteer support. In contrast, a food bank serves as a centralized organization that supplies food to pantries and other agencies that deliver assistance at the community level [12]. With a mission to alleviate or reduce food insecurity, food banks and pantries can play a crucial role in supporting individuals and families having difficulty accessing food. For example, in the United States, more than 50 million people have received food from the charitable food system, including food banks, pantries, and other community assistance programs [11]. In Canada, 66% of those experiencing food insecurity turn to local food banks for emergency food aid [13]. A systematic review of 35 global studies found that in order for food banks to improve food security, they require adequate operational resources, the availability of perishable foods, and the ongoing identification of client needs [14]. Food banks and pantries, however, face numerous operational challenges that could be mitigated by applying AI technologies. Together, food banks and pantries serve as central hubs in the charitable food system and food assistance network. Food banks store large quantities and varieties of rescued, donated, purchased, and government-provided food items and redistribute them to local community-based agencies, such as food pantries, where individuals and families can access food directly [15]. Despite their importance, food banks and pantries encounter significant challenges in redistributing donated food equitably and efficiently to advance their mission of reducing food insecurity [16,17]. These challenges include the high volume and variability of donations, the need for data-driven decision-making, and insufficient resources, including personnel and technology, to sort, take inventory of, and manage resources [1,17,18,19]. AI technologies offer immense potential to address these challenges by enhancing operations through providing data-driven insights, optimizing resource allocation, improving distribution efficiency, and minimizing waste.

However, compared to other areas of the food sector, such as agriculture and food industries [3,20,21,22], as well as other sectors such as healthcare, education, transportation, and public administration [23,24,25,26], AI applications in food banks and pantries remain underexplored. Thus, there is a pressing need to review the current literature on AI applications in food banks and pantries to evaluate its quality, identify existing gaps, and establish a foundation for targeted and practical future applications. Thus, this review work addresses the following three research questions:

(1) Which AI techniques are commonly employed to address operational challenges in food banks and pantries, and how effective are they?
(2) What are the methodological and ethical issues facing AI applications in food banks and pantry services?
(3) What policy and practical implications arise from using AI to improve the operations of food banks and pantries?

## 2. Methods

This systematic review was conducted following the Preferred Reporting Items for Systematic Reviews and Meta-Analyses (PRISMA) guidelines [27], and it was publicly registered on the Open Science Framework (OSF) platform for transparency purpose under the registration code (https://doi.org/10.17605/OSF.IO/25ZB9). An Institutional Review Board study review was not relevant as the research activities did not involve human subjects in their design or execution [28].

### 2.1. Inclusion and Exclusion Criteria

Studies that met all the following inclusion criteria were included in this review: (1) any empirical study design, including experimental, observational, or mixed-method designs; (2) a focus on the application of AI in food pantries and food banks, specifically in the public sector, not the private sector; (3) peer-reviewed publications; (4) studies published before 15 April 2025; and (5) articles written in English. Specifically, to ensure the comprehensive coverage of the relevant literature, we included all empirical research, regardless of the study design and publication year.

Studies that met any of the following criteria were excluded: (1) studies that did not involve or examine the use of AI applications in food pantries or food banks; (2) studies conducted solely in private sector settings without a focus on public sector food distribution systems; (3) studies that did not provide empirical research findings (e.g., letters, editorials, study protocols, conference proceedings, books, or review articles); (4) articles written in languages other than English.

### 2.2. Search Strategy

A keyword search was performed across 11 electronic bibliographic databases: PubMed, Web of Science, Scopus, MEDLINE, APA PsycArticles, APA PsycInfo, CINAHL Plus, EconLit with Full Text, Applied Science & Technology Full Text (H.W. Wilson), Family & Society Studies Worldwide, and SocINDEX with Full Text. The selection of the databases captured a wide breadth of research at the intersection of artificial intelligence, community health, and social services. The inclusion of PubMed, MEDLINE, and CINAHL Plus ensured we identified studies on nutrition, public health impacts, and service delivery models in food bank and pantry services. Web of Science, Scopus, and Applied Science & Technology Full Text covered core AI and machine learning research, including algorithm development and real-world applications. The inclusion of APA PsycArticles, APA PsycInfo, Family & Society Studies Worldwide, and SocINDEX helped us locate work on volunteer management, user behavior, and organizational psychology in food assistance programs. Finally, EconLit with Full Text contributed research on economic analyses of food allocation, cost–benefit evaluations, supply–demand matching, and other operational efficiency factors. A sample algorithm used for the search of PubMed can be found in Appendix A. The search algorithm included all possible combinations of keywords from two conceptual groups:

(1) “food bank”, “food banks”, “food pantry”, “food pantries”, “food shelf”, “food shelves”, “food distribution”, “food redistribution”, “food service”, “food services”, “community food program”, “community food programs”, “hunger relief organization”, “hunger relief organizations”, and “food assistance”;
(2) “artificial intelligence”, “computational intelligence”, “machine intelligence”, “computer reasoning”, “machine learning”, “deep learning”, “neural network”, “neural networks”, and “reinforcement learning”.

The search strategy targeted the title (TI), abstract (AB), and subject heading (SU) fields. The titles and abstracts of the articles identified through the keyword search were screened against the study selection criteria. Potentially relevant articles were retrieved for a full-text evaluation. Two researchers independently screened the titles and abstracts and identified studies for the full-text review. Cohen’s kappa (κ = 0.74) was calculated to assess the inter-rater agreement, and discrepancies were resolved through discussion. Two researchers reviewed the full text of articles identified from the title and abstract screening, and the final sample of included studies was jointly determined.

### 2.3. Study Screening

We used a two-stage selection process. First, duplicates were removed using EndNote. In the first stage, two reviewers reviewed the study objectives, research questions, and detailed inclusion and exclusion criteria, completed a pilot screening test by independently screening a small sample of titles and abstracts, and discussed discrepancies to align their understanding of the inclusion/exclusion criteria. Next, they independently screened all titles and abstracts, resolving any conflicts through discussion with the research team; studies deemed eligible or unclear in terms of eligibility at this stage moved on to the second round (i.e., full-text review). In the second stage, the two reviewers independently assessed full texts, with any remaining disagreements settled by consulting the research team.

### 2.4. Data Extraction

We used a standardized data extraction form to gather key information on each included study, including the authors, publication year, country/region, sample size, data source, study purpose, operational stage, AI models, validation methods, performance metrics, results, and policy or intervention implications. Given the substantial heterogeneity in the study designs and outcome measures, conducting a meta-analysis was not feasible. Instead, we synthesized the common themes and findings of the included studies through a narrative summary (see Table 1 and Table 2).

### 2.5. Quality Assessment

We conducted quality assessments for all the included studies using the Quality Assessment Tool for Observational Cohort and Cross-Sectional Studies developed by the National Institutes of Health [34]. This tool evaluates studies based on 14 questions, assigning a score of one for a “yes” response and zero otherwise. Two researchers independently performed the quality assessments, and a total score was calculated to reflect the overall study quality.

## 3. Results

### 3.1. Study Selection

**Figure 1:**
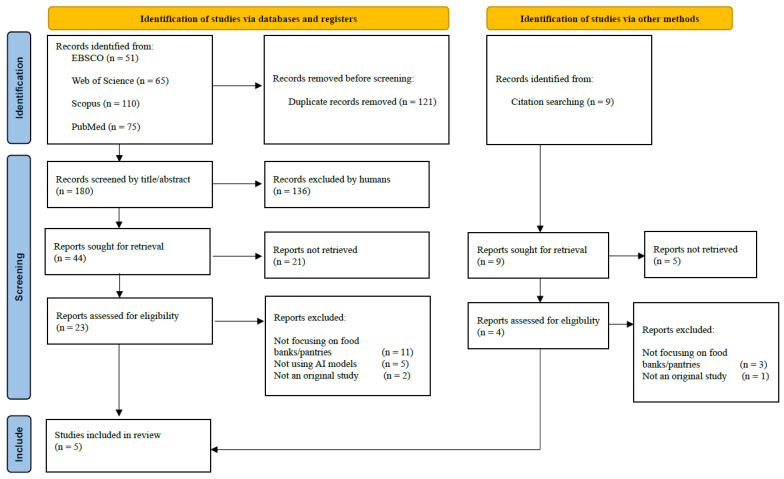
PRISMA study selection flow chart and search results [27]. Page MJ et al. BMJ 2021;372:n71. DOI: 10.1136/bmj.n71.

Figure 1 displays the flow of the study selection process. A total of 301 articles were identified through a keyword search across 11 databases managed by four major academic platforms: 51 articles from EBSCO, 65 articles from Web of Science, 110 articles from Scopus, and 75 articles from PubMed. After removing duplicates, 180 unique articles entered title and abstract screening, of which 121 were excluded. After the title and abstract screening, 23 articles were retrieved for a full-text review against the study selection criteria. Of these, eighteen articles were excluded for the following reasons: eleven studies did not focus on food banks or pantries, five did not employ AI models, and two were not empirical research. Five studies met the inclusion criteria and were chosen as the included papers.

### 3.2. Summary of Selected Studies

Table 1 presents the key characteristics of the studies included in this systematic review. The studies were conducted within a limited range of geographic contexts, including two in North Carolina, USA [29,32]; one in Ohio, USA [30]; one in southwest London, UK [31]; and one in an unknown location. The samples ranged widely from comparatively small participant surveys (i.e., 544 participants) [31] to extensive operational datasets (e.g., 17,555 food collection records and 15,000 donation records) [31,32,33]. Three operational stages of food bank and pantry systems were covered. The most frequently studied stage was food donation [31,32,33], followed by food collection [29] and food distribution [30]. The data types included food bank records, participant surveys, USDA datasets, and food images. Notably, articles were published in the fields of enterprise management, planning sciences, public sector marketing, and expert systems.

The included studies indicated several study purposes related to food bank/pantry operations. For instance, Brock and Davis [29] evaluated methods to estimate the donatable food availability at supermarkets, while Bennett et al. [31] explored donor attitudes and motivations. Sharmila et al. [32] optimized food donation allocation across a multi-warehouse hunger relief network, and Sucharitha and Lee [30] focused on predicting food demand and accessibility patterns to improve operational efficiency. The outcomes varied across studies, including enhanced food availability estimates [29], insights into donor perceptions [31], predicted donation quantities [32], optimized food demand prediction accuracy [30], and improved food quality assessment and warehousing management [33].

Each of these studies uniquely advanced AI-driven food bank operations. Brock and Davis [29] tested a multi-layer perceptron neural network which could yield more accurate in-kind donation forecasts and enable the cost-efficient routing of collection vehicles. Sucharitha and Lee [30] showed that the soft clustering of demand patterns using a Gaussian mixture model cuts forecasting errors and can improve inventory management and redistribution planning. Bennett et al. [31] provided the first quantitative mapping of individual donor motivations in the UK using structural topic modeling, guiding more effective engagement strategies. Sharmile et al. [32] integrated fine-grained supply forecasts into an equitable allocation model, cutting the forecast error by up to 48% and systematically identifying underserved areas, and Wu and Tai [33] introduced the first end-to-end AI pipeline achieving about 90% precision in spoilage detection and markedly improving storage–space utilization with lower computational costs.

Table 2 provides an overview of the AI models employed, the key findings, and the policy implications of the included studies. Four of the five studies utilized structured data machine learning algorithms, while one applied text data natural language processing (NLP) through structural topic modeling (STM) [31]. Two of the four machine learning studies employed neural network models (i.e., a multi-layer perceptron neural network [29] and convolutional neural network [33]). The remaining two studies predominantly used unsupervised machine learning methods, such as K-means clustering [32], and supervised learning techniques, including a random forest (RF) and Bayesian additive regression trees (BARTs) [33]. Different validation methods were applied, with handout splits being the most common approach, used in four studies (e.g., 60-40 or 70-30 training–testing splits) [29,30,32,33], while k-fold cross-validation was used in one study [32]. Scenario-based testing was exclusively employed in reinforcement learning approaches [33].

Regarding performance metrics, three studies utilized traditional metrics like the mean square error, mean absolute error, and root mean square error to evaluate the predictive performance [29,30,32]. Sharmile et al. [32] also adopted the mean absolute percentage error to assess supply chain flexibility. Bennett et al. [31] focused on interpretability, using Stone–Geisser Q^2^ values for structural topic modeling. Wu and Tai [33] assessed precision using the mean average precision, and bootstrapping methods were employed to validate the model’s robustness and reliability [30].

Overall, AI-driven methods showed tangible benefits in different operational stages for food services. To improve the food donation process, Bennet et al. [31] provided insights into donor attitudes, enabling targeted engagement strategies; Sharmile et al. [32] reported improvements in supply chain flexibility and coordination by optimizing the allocation of food donations across multi-warehouse networks; and Wu and Tai [33] enhanced warehousing management and the quality assessment of donated food. One study focused on predicting the food availability at supermarkets for the collection process, which enabled better transportation cost management and logistical efficiency [29]. In the distribution stage, Sucharitha and Lee [30] achieved robust demand forecasting that facilitated the more equitable distribution of resources to underserved areas. These detailed results underscore how AI models, including supervised, unsupervised, reinforcement learning, and NLP techniques, substantially enhance the forecasting accuracy, supply allocation fairness, demand estimation, quality assessment, and donor engagement insights across food bank and pantry operations.

### 3.3. Quality Assessments of Included Studies

Table 3 displays the NIH Quality Assessment Tool for Observational Cohort and Cross-Sectional Studies [34]. All five included studies showed strengths in terms of their clearly stated research objectives and well-defined study populations. The outcome measures were consistently valid and reliable across all studies. However, the participation and attrition rates after baseline were not reported in any study, and only one study provided a sample size justification [31]. While exposure measures were consistently implemented, only two studies assessed exposures more than once over time [30,33].

## 4. Discussion

This study systematically reviewed the emerging empirical evidence on AI applications in food bank and pantry services, examining their effectiveness, potential benefits, and challenges. The five included studies adopted various AI methods such as neural networks, clustering, random forests, Bayesian trees, and natural language processing. Although limited by their geographic contexts, the findings indicate that AI has great potential to optimize food donation, collection, and distribution processes, improving efficiency and equity in food bank and pantry efforts.

Although AI has yet to be fully integrated into food banks and pantries, AI has displayed its ability to optimize supply chains, improve distribution efficiency, and enhance resource allocation in various food security-related sectors [35]. The greater adoption of AI in relation to food bank and food pantry services could follow similar relevant advancements, such as AI-driven predictive analytics that have successfully forecast the food demand in broader distribution networks, minimizing shortages and food waste [36,37,38]. Similarly, AI-powered logistics systems have enhanced transportation efficiency by optimizing delivery routes and reducing operational costs [39], a model that food banks and pantries could adopt to ensure more equitable food distribution. AI-powered chatbots and recommendation automation are already used in public service programs to assist vulnerable individuals, and integrating these technologies into food assistance programs could help marginalized communities navigate food bank services, access eligibility information, and receive personalized support. Translating these advancements for food assistance systems has the potential to reduce disparities, improve access to nutritious food, and increase consistency and quality for households and communities disproportionately experiencing food insecurity.

Despite the promising advancements, this review identified several methodological and practical challenges. Among the 301 initial articles, only 5 studies met the inclusion criteria, highlighting a current scarcity of research on AI applications in food bank and pantry operations. This might be due to four interrelated barriers: (1) resource constraints, which refer to the tight budgets, volatile volunteer and staff turnover, and heavy reliance on in-kind and monetary donations [17,40] that make it challenging to fund AI infrastructure, data platforms, and specialized personnel; (2) data challenges, meaning fragmented, inconsistent, or incomplete records [41] that prevent the training and validation of robust models and discourage investment in experimental technologies; (3) operational heterogeneity, representing the wide variation in processes, scales, and resource availability [17] across food banks and pantries, which complicates the development of generalizable AI solutions; and (4) institutional barriers such as client privacy concerns, limited partnerships with academic or technology institutions, and competing organizational priorities that slow the translation of AI prototypes into practice. Overcoming these obstacles will require sustained funding for digital capacity building, cross-sector collaborations to standardize data governance and collection, and the creation of open-source, low-resource AI toolkits tailored to the contexts of food banks and pantries, paving the way for scalable, impactful AI interventions to reduce food insecurity.

While all five studies clearly defined their research objectives and outcome measures, none reported participation rates, that outcome assessors were blinded to the exposure status of the participants, or the loss rate after baseline, and only one provided a sample size justification [31]. Furthermore, exposure assessments were inconsistently implemented, with only two studies conducting repeated exposure measurements [30,32]. Although the included studies showed the use of AI models across different food bank and pantry services processes, none addressed critical data limitations, such as data representativeness, the participation rate of eligible subjects, and the attrition rate after baseline.

In addition to methodological challenges, the application of AI in food bank and pantry operations faces practical barriers. For example, ethical challenges, such as model bias and fairness in resource allocation, also present risks [42,43,44]; AI models trained on unrepresentative datasets may reinforce existing disparities, while decisions driven solely by algorithmic efficiency could inadvertently overlook equity considerations [45,46,47]. None of the included papers discussed the potential ethical challenges of adopting these AI models. Moreover, user engagement barriers, including language and cultural differences, social stigma, and limited digital literacy, could undermine the accessibility and usability of AI-powered systems, particularly for diverse and underserved client populations [48,49].

Future research should systematically advance AI applications in food bank and pantry settings by first addressing fundamental methodological gaps through developing standardized evaluation protocols, rigorous sampling methods, and longitudinal study designs to establish more substantial evidence for AI’s effectiveness. Building on this methodological foundation, research must create or translate appropriate AI ethical frameworks for food banks and pantries to ensure data representativeness and algorithmic fairness in resource allocation decisions, particularly given the vulnerable population which uses food banks and pantries. Finally, given the limited research on user engagement, future work should investigate culturally responsive AI system designs that address diverse user needs, organizational and individual digital literacy barriers, and strategies to reduce the stigma associated with food assistance services. These areas remain largely unexplored in the current literature but are crucial for successful AI implementation and the practical and responsible use of AI in food bank and pantry operations.

This study has important implications for practical operations and policy design. The opportunity for the use of AI in food bank and pantry settings is immense in terms of the scope of these programs and the amount of food distributed and number of individuals served and as a setting for additional nutrition and food security-based interventions at the community level [15]. From an operational perspective, AI provides the opportunity to revolutionize food assistance systems, making the food service operation process more data-driven, efficient, and responsive. Policymakers and philanthropy should prioritize providing support for AI adoption in food assistance programs by allocating funding and developing infrastructure and regulatory frameworks to promote equitable AI implementation. Special attention should be given to supporting AI applications in low-income and rural areas, where food insecurity is the most prevalent and the need for efficient food assistance systems is the most significant. Meanwhile, governments can establish data-sharing protocols and governance models that encourage cross-sector collaboration while protecting user privacy and ensuring ethical AI use. Food banks and pantries can also explore lower-cost AI solutions and bolster their own internal data policies, practices, and procedures to achieve greater adoption and scalability given resource constraints. In addition to examining the advancement of AI technologies used in food banks and pantries, research can also seek to better understand data practices and AI approaches in the charitable food system through Implementation Science.

This review represents the first study to systematically synthesize the empirical evidence on AI applications in food banks and pantry services. It made several contributions: (1) it centered on the field of public sector-oriented food services, specifically food bank and pantry operations, rather than focusing on agriculture or business-centered food services; (2) it examined the diverse processes where AI is applied, including food collection, donation, and distribution; (3) it systematically investigated how studies addressed or considered ethical AI practices and policy implications; and (4) it derived actionable recommendations from the study findings to guide future applications of AI in food services and operations.

However, several limitations of this review should be acknowledged. First, selection and publication biases may have arisen from our decision to include only peer-reviewed journal articles in English. This excluded conference proceedings, white papers, policy reports, and non-English publications, all of which may report emerging AI applications or null results that never reach indexed journals. Second, our narrow focus on empirical studies on AI in food bank and pantry contexts led to there being only five included papers, limiting the generalizability of our findings and raising concerns about small-study effects. Third, we did not formally assess the risk of bias within the included studies. None reported their participant eligibility rates, attrition, assessor blinding, or sample size justifications, and ethical considerations such as algorithmic fairness and data privacy went unaddressed, all of which may have introduced bias into their reported outcomes. Fourth, heterogeneity across the study designs, AI methods, and performance metrics precluded meta-analysis and made it challenging to compare effect sizes directly. Finally, while comprehensive, covering 11 databases, our search strategy may still have missed relevant work from related sectors and non-academic outlets.

## 5. Conclusions

This study systematically reviewed empirical evidence on AI applications in food banks and pantry services. We found that AI models used in food bank and pantry settings span supervised learning, unsupervised learning, and natural language processing, demonstrating clear gains in donation forecasting, demand estimation, ensuring allocation equity, and understanding donor motivations. However, significant methodological and ethical gaps remain. Future work should broaden the evidence base across food bank and pantry contexts, establish standardized, high-quality data and governance protocols, ensure ethical safeguards and bias mitigation practices, and foster practitioner co-design. Meanwhile, it is equally important to develop accessible AI toolkits and perform real-world impact evaluations to ensure that technology investments translate into effective reductions in food insecurity.

## Figures and Tables

**Table 1:** Characteristics of the included studies.

Authors (Year)	City, Country	Sample Size	Data Source	Operational Stage	Study Purpose	Outcome	Main Contributions
Brock and Davis (2015) [29]	North Carolina, US	17,555 food collection records	Food Bank of Central and Eastern North Carolina (FBCENC)	Food collection	Evaluate four approximation methods regarding their ability to estimate food availability at supermarkets	Food availability at supermarkets	Enabled more accurate in-kind donation estimates and cost-efficient routing for food collection vehicles
Sucharitha and Lee (2022) [30]	Ohio, US	15,000 food donation records	Greater Cleveland Food Bank and USDA data	Food distribution	Understand and predict food demand and accessibility patterns for food banks and food assistance programs to help organizations optimize their operations and distribution of food aid to people in need	Prediction accuracy for food demand	Showed that soft clustering of demand patterns yields higher error reduction, improving inventory and redistribution planning
Bennett et al. (2023) [31]	Southwest London, UK	544 participants	Supermarket exit survey	Food donation	Investigate the attitudes and motivations of individuals donating to food banks	Perceptions of food bank donors	Provided the first quantitative mapping of UK individual donor motivations
Sharmile et al. (2024) [32]	North Carolina, US	NA	Food Bank of Central and Eastern North Carolina (FBCENC)	Food donation	Predict and optimize the fair allocation of in-kind food donations in a multi-warehouse hunger relief supply chain network	Quantity of food donations received per month; meals served per person in need (MPIN)	Integrated fine-grained supply forecasts into an equitable allocation model, cutting the forecast error and systematically identifying underserved areas
Wu and Tai (2024) [33]	NA	4784 images of food	Traditional markets, supermarkets, and the Internet	Food donation and storage	Improve the inbound logistics of food banks, specifically in the areas of food quality assessment and warehousing management	Quality assessment of donated food (mean average precision); optimization of storage decisions (storage–space ratio)	Introduced the first end-to-end AI pipeline for food banks’ inbound logistics, achieving high precision for spoilage detection and showing that RL can markedly improve storage–space utilization with lower computational costs

**Table 2:** AI models, key findings, and policy implications of the included studies.

Authors (Year)	Models	Validation Methods	Performance Metrics	Results	Policy/Intervention Implications
Brock and Davis (2015) [29]	Multi-layer perceptron neural network (MLP-NN)	Handout method: 60% training and 40% test set split	Mean square error (MSE), mean absolute error (MAE), and coefficients of determination (R^2^)	The MLP-NN models were superior to the SM Average model, SMWH Average model, and Multiple Linear Regression model, both in terms of prediction accuracy and the impacts on transportation costs.	NA
Sucharitha and Lee (2022) [30]	Gaussian mixture model (GMM), Generalized Linear Model (GLM), Generalized Additive Model (GAM), Multivariate Adaptive Regression Splines (MARSs), random forest (RF), and Bayesian additive regression trees (BARTs)	k-fold cross validation Handout method: 70% training and 30% test set split	Accuracy, MSE, MAE, and adjusted R-squared	The two-stage prediction model yielded an accuracy of up to 82% in predicting the individual and family food demand, and the results also suggested the need to redistribute food assistance to underserved areas.	NA
Bennett et al. (2023) [31]	Open-ended structural topic modeling (STM)	Bootstrapping	Stone–Geisser Q^2^ values	STM identified three key perceptions among donors (deservingness, vulnerability, and victimhood) and non-donors (mendicant, undeserving, and apathy).	Launch targeted promotional campaigns Leverage religious and community channels Create accessible donation options and appeal to emotional benefits Address misconceptions about poverty
Sharmile et al. (2024) [32]	Machine learning models, including K-means clustering algorithm	Handout method, but without specifying percentage for training and test set split	Mean absolute percentage error (MAPE), root mean square error (RMSE), MAE	Higher supply chain flexibility and coordination enabled more equitable distribution of donated supplies.	NA
Wu and Tai (2024) [33]	Convolutional neural network (CNN) and reinforcement learning approach	Handout method: 80% training and 20% test set split Tested reinforcement learning models in different scenarios	Mean average precision (MAP)	The CNN-based approaches for food quality assessment and warehousing management exceeded the expectations of food bank managers, achieving positive disconfirmation when evaluated through the lens of expectation– confirmation theory.	NA

**Table 3:** Quality assessment * tool for observational cohort and cross-sectional studies designed by National Heart, Lung, and Blood Institute.

	Brock and Davis (2015) [29]	Sucharitha and Lee (2022) [30]	Bennett et al. (2023) [31]	Sharmile et al. (2024) [32]	Wu and Tai (2024) [33]
1. Was the research question or objective in this paper clearly stated?	Yes	Yes	Yes	Yes	Yes
2. Was the study population clearly specified and defined?	Yes	Yes	Yes	Yes	Yes
3. Was the participation rate of eligible persons at least 50%?	NA	NR	NR	NR	NR
4. Were all the subjects selected or recruited from the same or similar populations (including during the same time period)? Were the inclusion and exclusion criteria for being in the study prespecified and applied uniformly to all participants?	NA	Yes	Yes	Yes	NA
5. Were sample size justifications, power descriptions, or variance and effect estimates provided?	NA	No	Yes	No	No
6. For the analyses in this paper, was the exposure(s) of interest measured prior to the outcome(s) being measured?	NA	Yes	Yes	Yes	Yes
7. Was the timeframe sufficient so that one could reasonably expect to see an association between the exposure and outcome if it existed?	NA	Yes	NA	Yes	NR
8. For exposures that can vary in their amount or level, did the study examine different levels of the exposure as related to the outcome (e.g., categories of exposure or exposure measured as a continuous variable)?	NA	Yes	No	Yes	No
9. Were the exposure measures (independent variables) clearly defined, valid, reliable, and implemented consistently across all study participants?	Yes	Yes	Yes	Yes	Yes
10. Was the exposure(s) assessed more than once over time?	NA	Yes	No	Yes	CD
11. Were the outcome measures (dependent variables) clearly defined, valid, reliable, and implemented consistently across all study participants?	Yes	Yes	Yes	Yes	Yes
12. Were the outcome assessors blinded to the exposure status of participants?	NA	No	No	No	No
13. Was the loss to follow-up after baseline 20% or less?	NA	NR	NA	NA	NA
14. Were key potential confounding variables measured and adjusted statistically concerning their impact on the relationship between the exposure(s) and outcome(s)?	NA	Yes	Yes	Yes	Yes
Total score	4	10	8	10	6

* This study’s quality assessment tool was adapted from the National Institutes of Health’s Quality Assessment Tool of Observational Cohort and Cross-Sectional Studies [34]. For each criterion, studies were rated “Yes”, “No”, or other (i.e., CD: cannot determine; NR: not reported; NA: not applicable).

## Data Availability

The sample search algorithms used to extract the papers can be found in Appendix A.

## Appendix A. Sample Search Algorithm Used in PubMed

(“artificial intelligence”[mh] OR “artificial intelligence”[tiab] OR “computational intelligence”[tiab] OR “machine intelligence”[tiab] OR “computer reasoning”[tiab] OR “machine learning”[tiab] OR “deep learning”[tiab] OR “neural network”[tiab] OR “neural networks”[tiab] OR “reinforcement learning”[tiab]) AND (“food bank”[tiab] OR “food banks”[tiab] OR “food pantry”[tiab] OR “food pantries”[tiab] OR “food shelf”[tiab] OR “food shelves”[tiab] OR “food distribution”[tiab] OR “food redistribution”[tiab] OR “food service”[tiab] OR “food services”[tiab] OR “food services”[mh] OR “community food program”[tiab] OR “community food programs”[tiab] OR “hunger relief organization”[tiab] OR “hunger relief organizations”[tiab] OR “food assistance”[tiab] OR “food assistance”[mh])
